# Determining Clinically-Viable Biomarkers for Ischaemic Stroke Through a Mechanistic and Machine Learning Approach

**DOI:** 10.1007/s10439-022-02956-7

**Published:** 2022-04-01

**Authors:** Ivan Benemerito, Ana Paula Narata, Andrew Narracott, Alberto Marzo

**Affiliations:** 1 INSIGNEO Institute for In Silico Medicine, The University of Sheffield, Sheffield, UK; 2 Department of Mechanical Engineering, The University of Sheffield, Sheffield, UK; 3 Department of Neuroradiology, University Hospital of Southampton, Southampton, UK; 4 Department of Infection, Immunity and Cardiovascular Disease, The University of Sheffield, Sheffield, UK

**Keywords:** Leptomeningeal collateral, Ischaemic stroke, Gaussian process emulator, Sensitivity analysis, Biomarker, Cardiovascular modelling, Brain circulation, Wave propagation

## Abstract

Assessment of distal cerebral perfusion after ischaemic stroke is currently only possible through expensive and time-consuming imaging procedures which require the injection of a contrast medium. Alternative approaches that could indicate earlier the impact of blood flow occlusion on distal cerebral perfusion are currently lacking. The aim of this study was to identify novel biomarkers suitable for clinical implementation using less invasive diagnostic techniques such as Transcranial Doppler (TCD). We used 1D modelling to simulate pre- and post-stroke velocity and flow wave propagation in a typical arterial network, and Sobol’s sensitivity analysis, supported by the use of Gaussian process emulators, to identify biomarkers linked to cerebral perfusion. We showed that values of pulsatility index of the right anterior cerebral artery > 1.6 are associated with poor perfusion and may require immediate intervention. Three additional biomarkers with similar behaviour, all related to pulsatility indices, were identified. These results suggest that flow pulsatility measured at specific locations could be used to effectively estimate distal cerebral perfusion rates, and ultimately improve clinical diagnosis and management of ischaemic stroke.

**Supplementary Information:**

The online version contains supplementary material available at 10.1007/s10439-022-02956-7.

## Introduction

Ischaemic stroke (IS), the most common cerebrovascular disease and the second cause of death worldwide, constitutes a significant burden on the healthcare systems of many countries.^15^ It is caused by a thrombus which obstructs a major vessel and limits the amount of blood that perfuses the districts downstream of the occlusion, causing tissue death if the perfusion is not swiftly restored.^29^

Several collateral pathways provide alternative perfusion routes in case of occlusion of a major vessel. The primary one, the Circle of Willis (CoW), provides an alternative perfusion route in obstructions of the internal carotid artery.^9,33^ In case of occlusions of the proximal middle cerebral artery (MCA), which accounts for the majority of all stroke cases,^41^ continuity of perfusion is provided by a network of smaller vessels, the pial or leptomeningeal anastomoses (LMAs). LMAs connect the MCA with posterior and distal anterior cerebral arteries (PCA and ACA2, respectively).^9,36^ This compensation mechanism is an important determinant of post-ischaemic tissue fate,^2^ but its effectiveness is highly dependent on the status of the LMAs and their ability to carry sufficient amounts of blood and metabolites.^18^ It has been observed that patients with effective collateral circulation recover better than patients with poor LMAs.^11^ Imaging methods, for example contrast and perfusion MR and CT, are commonly used in the diagnosis of IS and can evaluate the degree of collateralisation^8,33^ and perfusion impairment. However, they are expensive and require the injection of a contrast medium in the blood stream. Additionally, they only offer a snapshot in time of the patient’s condition and do not allow for continuous monitoring.^22^

Transcranial Doppler ultrasound (TCD) is not able to detect a signal from small vessels such as the LMAs but can be used to indirectly confirm the presence of IS and LMA collateralisation^8,33^ through non-invasive measurement of intracranial blood velocities and the evaluation of features of the velocity waveforms in large vessels such as ACA1 (proximal anterior cerebral artery), ACA2, PCA and MCA.^6,8,38^ Despite being used to evaluate the recanalisation state after treatment, TCD does not provide a quantitative measure of the degree of tissue perfusion for preoperative planning.^22^ The identification of velocity-based biomarkers of perfusion that could be measured via TCD would allow first responders to route IS patients along the most appropriate clinical pathway in a timely manner.^46^

Computational models of the CoW can help understanding the mechanics of stroke and collateralisation and have been used in a variety of scenarios. The use of 3D approaches is popular^13,21^ but poses challenges for domain definition, determination of parameters and required computational resources. 1D models allow either steady or transient simulations of larger vascular networks in shorter time frames,^30^ can incorporate nonlinear material properties,^5^ and have been used to study the brain circulation in physiological^16,44^ and pathological conditions, both alone^1,32^ or integrated with data driven techniques.^24,40^ Only recently have researchers started to include the pial circulation into their models,^25,27,28^ rarely proposing methodologies for clinical exploitation of their findings.

In this study we have a twofold aim, which we pursue through a hybrid mechanistic-statistical approach. The first aim is the development and validation of a 1D computational model of the LMAs to improve our mechanistic understanding of the influence of stroke on blood flow. The second aim is the identification of biomarkers for distal perfusion that could be computed from TCD measures routinely performed in a clinical setting. We employ Gaussian process emulators for the analysis of arterial blood velocities,^24^ and Sobol sensitivity analysis^10,24^ for the identification of biomarkers. This process recognises the potential variability of anatomical parameters within the patient population, and the effects of this on results variability. We will show that the model captures the fundamental role of LMAs during IS and that it is possible to identify a biomarker that could inform clinical management of IS from routinely performed clinical measurements.

## Materials and Methods

We first present the mechanistic model of the LMA circulation and describe its use for simulating healthy and stroke scenarios. Then, we describe the statistical model and introduce Gaussian Process Emulators (GPEs), for emulating the behaviour of the brain circulation model, and Sobol’s sensitivity analysis (SSA), for model reduction and biomarker identification.

### Mechanistic Baseline Model

**Figure 1:**
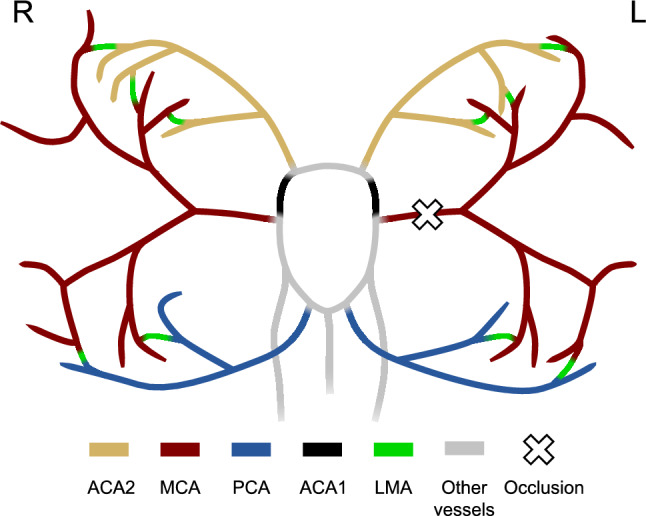
Portion of the network used in this study. ACA2 district is shown in yellow, MCA district in red, PCA district in blue, ACA1 in black, LMAs in green, other intracranial vessels in grey. Extracranial vessels are not shown. The cross identifies the occlusion location in the left MCA. The white circle indicates the locations where the velocities are measured to compute the biomarkers.

The network used in this study extends a subject generic model of the brain vasculature we have previously published and validated^24^ by further branching the PCA and the distal ACA (ACA2). The input parameters of the model (radii, length, and Young’s modulus of all vessels, windkessel parameters for the outlets and the inlet flow rate) were obtained from the literature. The original model, comprising the CoW and other extracranial arteries, is based on the model developed by Alastruey et al.^1^. Vessels branching from left and right MCA were derived from a study of Melis et al.^24^, while we used the ADAN model^7^ and a study from Phan et al.^27^ to obtain the data for branches of PCA and ACA2. Furthermore, we included the pial vessels that connect the anterior and posterior cerebral districts with the middle one. Figure 1 shows a diagram of the resulting network.

**Figure 2:**
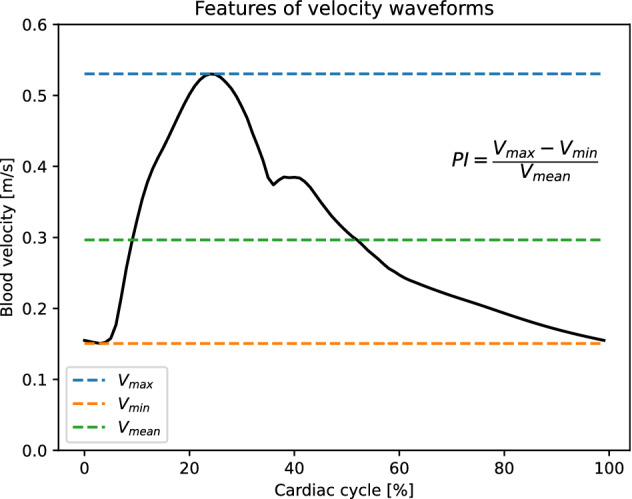
Typical velocity waveform from healthy ACA1 (black). Blue dotted line indicates its maximum value (peak systole), while orange and green dotted lines indicate the minimum(end-diastole) and time-averaged values respectively. The definition of pulsatility index (PI) for this waveform is shown on the right. The flow diversion (FD), not shown here, is computed as the ratio of the ipsilateral and contralateral average velocities.

The LMAs were modelled as 5 mm long and 400 μm^27,36^ wide direct connections between the major vessels.^27^ All arteries in the network were modelled as elastic 1D vessels able to deform in the radial direction.^1,35^ Arterial stiffness was location dependent, with larger and smaller vessels having lower and higher Young’s modulus respectively. The inflow of blood was prescribed at the ascending aorta as half a sinusoidal wave with peak amplitude of 485 mL/s and duration 0.3 s (systole). During the 0.7 s diastole the inlet flow rate was set to 0 mL/s.^1^ The peripheral parts of the vascular system were represented as three element RCR windkessel outlets.^1^ Values of resistance and compliance of individual outlets were computed by distributing the total peripheral vascular resistance and compliance to each outlet according to their area.^42^ The model described above constituted the baseline model. The complete representation of the model (mechanical parameters and network connectivity) is available in the Supplementary Material and as an online dataset^4^. The software openBF ^23^ was used to compute blood flow rate, velocity, and pressure in all vessels of the network over a full 1s cardiac cycle. The baseline model was used to simulate a healthy individual and a patient with complete ischaemic occlusion in the left MCA (Fig. 1). We regarded the MCA peripheral outlets as the brain region most directly affected by the stroke, and the total outflow in this region as the distal perfusion of interest in the study. We assessed the influence of the LMAs anatomy by comparing the post-stroke total outlet flow with the pre-stroke case. Furthermore, we computed flow diversion (FD) and pulsatility index (PI), as they are commonly used in the clinic to infer the presence of IS using TCD measurements.^17,33^ FD is defined as the ratio of the time-average blood velocity in a vessel in the ipsilateral side (same side) to the stroke to the average velocity in the contralateral (opposite side) vessel. Physiological values of FD in the ACA1 and PCA are around 1, while IS patients typically show FD>1.3.^17,33^ PI is defined as the difference between maximum and minimum blood velocity waveforms divided by its time-averaged value over a cardiac cycle.^6^ Patients with MCA stroke show PI<1.2 in the ACA and PCA^17,33^.Figure 2 shows a typical velocity waveform in the ACA1, and the equation used for calculation of PI.

### Gaussian Process Emulator

GPEs are statistical tools that can emulate the behaviour of complex nonlinear systems using only a limited number of model runs as training points.

We sampled the vessel radii and the outlet windkessel parameters of all the intracranial vessels, as it has been shown that these parameters are the most influential in determining blood velocities.^24^ This amounted to the analysis of 136 parameters. Using the Latin Hypercube method^10,24^ we sampled 1100 points from the 136-dimensional input parameter space by varying the variables within ±40% of their nominal value. These 1100 points were split into a training dataset of 900 points and a validation dataset of 200 points. All the 1100 points were simulated using openBF and used as inputs for a GPE with zero mean and squared exponential kernel.^24^ The emulator was trained to output the average perfusion (time-average over the cardiac cycle of the outlet flow in the left MCA region) and the candidate biomarkers that are detailed in the next section. The number of training points was chosen after performing a convergence study to guarantee a sufficient quality of the emulator fit on the validation dataset. Discrepancies between simulator and emulator were assessed through the mean average percentage error (MAPE),^10^ defined as

$${\text{MAPE}} = \frac{100\% }{{200}}\frac{1}{{\overline{{y_{s} }} }}\mathop \sum \limits_{n = 1}^{200} \left| {y_{s}^{n} - y_{e}^{n} } \right|$$

where $y_{s}^{n}$ and $y_{e}^{n}$ are the results from the $n$-th run of the simulator and emulator respectively, and $\overline{{y_{s} }}$. is the mean of the simulator output. The quality of the emulator fit was deemed acceptable when the MAPE was <7% on the entire validation dataset, in alignment with previous studies.^10^

With the emulator trained on the 136-dimensional space we generated the points for SSA as described in the next section

### Sensitivity Analysis for Model Reduction

Specific features of the velocity waveforms (minimum, time-average and maximum from left and right ACA1, ACA2 and PCA) were extracted from the 1100 openBF simulations and used to determine the candidate biomarkers, as listed in Table 1.

**Table 1:** List of potential biomarkers and their locations.

Candidate biomarker	Acronym
Flow diversion of ACA1	FD-ACA1
Flow diversion of PCA	FD-PCA
Flow diversion of ACA2	FD-ACA2
Pulsatility index of ACA1 left	PI-ACA1-L
Pulsatility index of ACA1 right	PI-ACA1-R
Pulsatility index of PCA left	PI-PCA-L
Pulsatility index of PCA right	PI-PCA-R
Pulsatility index of ACA2 left	PI-ACA2-L
Pulsatility index of ACA2 right	PI-ACA2-R
Product of pulsatility indices of ACA1, ACA2 and PCA left and right	PPI
Product of pulsatility indices of ACA1 left and right	PPI-ACA1
Product of pulsatility indices of PCA left and right	PPI-PCA
Product of pulsatility indices of ACA2 left and right	PPI-ACA2
Mean of pulsatility indices of ACA1, ACA2 and PCA left and right	meanPI

The candidate biomarkers in Table 1 were selected based on FD and PI and in alignment with established clinical protocols, where this type of measurements are routinely taken in the management of stroke patients.^17,33^

The effectiveness of biomarkers in capturing the extent of distal MCA perfusion was assessed through Sobol sensitivity analysis (SSA).^10,24^ SSA decomposes the output variance of a mathematical model and attributes it to the variance of individual inputs. The influence of an input on the outputs is quantified through the Sobol indices, with 0 and 1 corresponding to minimum and maximum influence respectively. A thorough exploration of the input space of a model with $N$ input parameters requires $O\left( {1000*N} \right)$ simulations,^24^ which is often computationally prohibitive using a mechanistic model. We generated 110000 points for sensitivity analysis using the statistical emulator: the number of points was selected to ensure convergence of the Sobol algorithm. Following this we identified the parameters with Sobol’s indices >0.1^10^ that defined the reduced space.

### Biomarker Identification

We restricted the use of the emulator to the reduced space, and generated an additional 110000 points to compute the Sobol’s indices for two scenarios of input parameter variability:

1. 40% variability on all the model parameters.
2. 10% variability on the ACA and PCA radii, 40% variability on all the remaining model parameters.

The first scenario corresponds to the case where the least information is known about the patient’s anatomy. The second scenario refers to the case where the radii of ACA and PCA are estimated using TCD to reduce uncertainty on these values.^14^ Finally, an output is regarded as a biomarker if it is sensitive to the same set of input parameters as the average distal perfusion.

### Correlation Analysis

To further characterise the identified biomarkers, we studied their correlation with the distal perfusion when the levels of uncertainty on the radii of ACA2 and PCA varied continuously from 10% to 40%. The uncertainty on all the other parameters was kept to 40%.

Sobol indices and correlation surfaces describe the strength of the relationship between two quantities but do not give explicit information on their mutual dependency. We generated a pool of 10000 virtual patients with 10% uncertainty on ACA2 and PCA and produced scatter plots of the perfusion against the biomarkers. From these relationships we identified biomarker thresholds to stratify patients into groups with perfusion below 50% and those with perfusion level above 50%. The 50% perfusion value was selected based on clinical evidence that prolonged regional brain perfusion lower than 50% is associated with cerebral damage and requires immediate intervention.^11,12^ Using this perfusion value, we subdivided the biomarker/perfusion plane into four quadrants. The upper left quadrant (A) correctly stratifies patients into the group requiring immediate intervention. The upper right one (B) includes patients incorrectly identified as needing urgent care. Patients in the lower left quadrant (C) are incorrectly classified as not needing priority treatment, while those in the lower right quadrant (D) are correctly classified by the biomarker. The optimization process identifies the value of the biomarker threshold that maximises the ratio

$$H = \frac{{\mathop \sum \nolimits_{i} A_{i} + D_{i} }}{{\mathop \sum \nolimits_{i} A_{i} + B_{i} + C_{i} + D_{i} }}$$

where $A_{i} , B_{i} ,C_{i}$ and $D_{i}$ denote the points belonging to A, B, C and D respectively. This approach guarantees that the error in predicting the level of perfusion using the given biomarker threshold is minimum over the entire population, and thus can be used as a threshold for clinical decision making.^19^

Furthermore, from the scatter plots it was possible to compute the probabilities that, for a given value of the biomarker, the patients exhibit a perfusion above 50%. This is computed as the ratio of points above 50% perfusion to the total number of points corresponding to an observed value of the biomarker.

## Results

### Mechanistic Baseline Model

**Figure 3:**
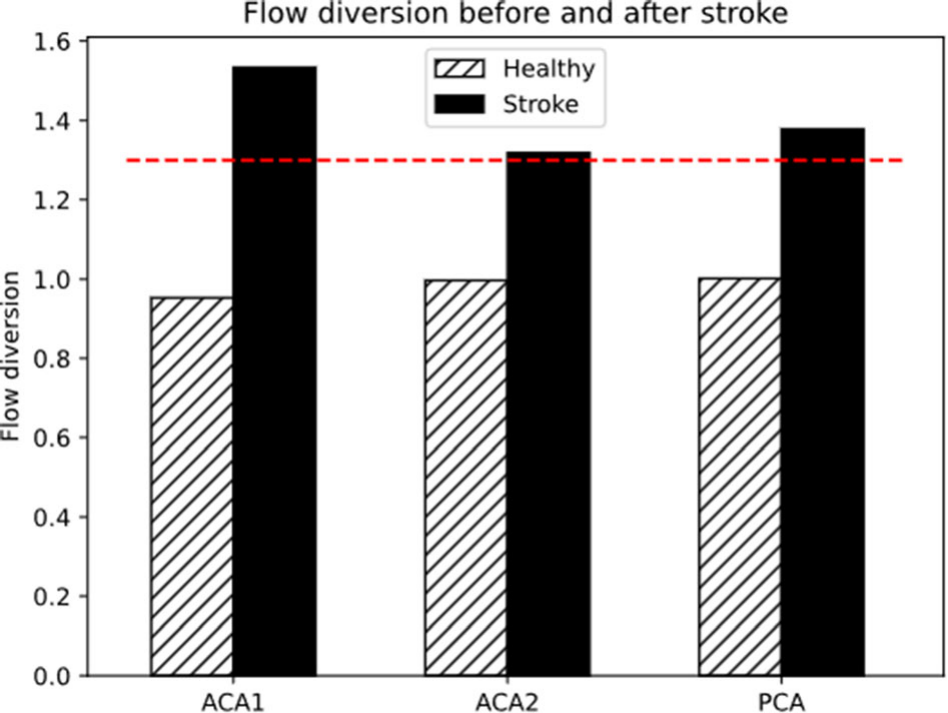
The flow diversion in ACA1, ACA2 and PCA increases after stroke (black bars) with respect to the healthy case (hatched bars) and reaches values above 1.3 (red dashed line), which is a sign of LMA collateralisation.

**Figure 4:**
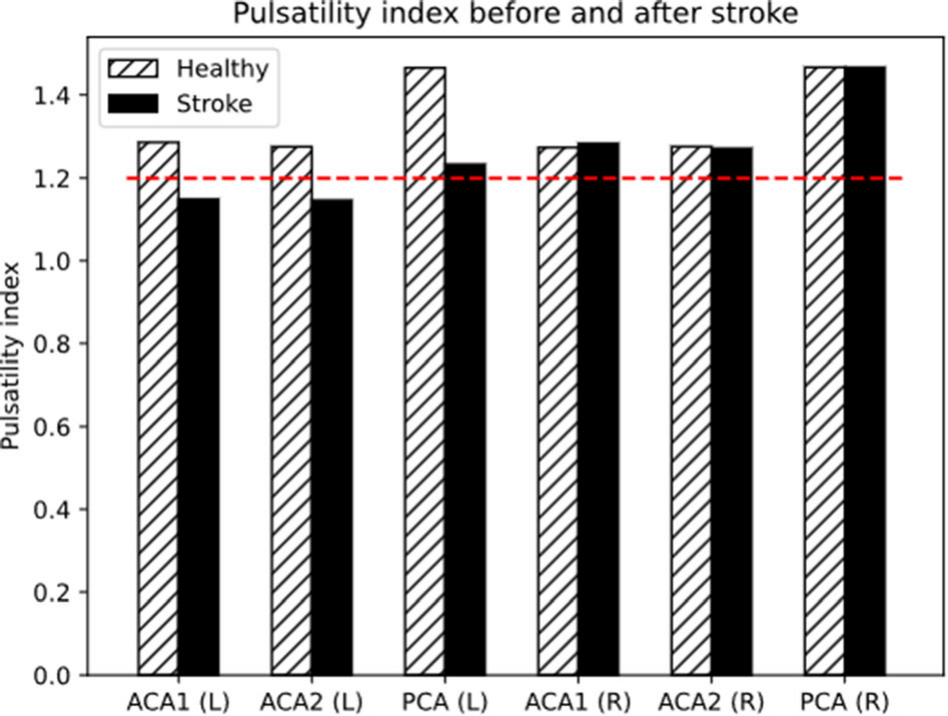
Following left MCA stroke (black bars) the pulsatility index of left ACA1, ACA2 and PCA decreases, while the right side is only minimally affected. White bars show the values in the healthy case, and the red dashed line indicates PI=1.2: values before 1.2 are signs of distal LMA collateralisation.

The simulation of a healthy individual presents a balanced circulation, with negligible differences in velocities, flowrate, and pressure between homologous vessels on the two sides of the brain, and values of flow diversion are 0.95, 0.99 and 1 in proximal ACA, distal ACA and PCA respectively (Fig. 3). Pulsatility indices in these vessels are 1.3, 1.3 and 1.48 on both right and left side, as shown in Fig. 4. The occlusion of left MCA causes a pressure drop from 70 to 53 mmHg in its distal region. The effect on ipsilateral ACA2 and PCA is smaller, with drop from 75 to 73 mmHg and from 80 to 77 mmHg respectively, but enough to generate a pressure gradient which forces blood from ACA2 and PCA into the LMAs. This blood is then distributed to the vessels of the MCA district downstream the occlusion. Figures 3 and 4 show the values of flow diversion and pulsatility index after the stroke. Average post-stroke MCA perfusion ranges from 57% to 79% (68±7 %), showing how LMAs act as collateral pathways. Alterations to blood flow on the contralateral side are negligible.

### Model Reduction and Biomarker Identification

Amongst the 136 parameters of the full model we identified 11 that had a major influence (Sobol’s index>0.1) on the determination of candidate biomarkers and distal perfusion. These parameters are the radii of left and right ACA1, ACA2 and PCA, the radius of LMA, the resistive part of the windkessel elements in left and right ACA2 and PCA districts. Detailed results are available in the Supplementary Material.

**Figure 5:**
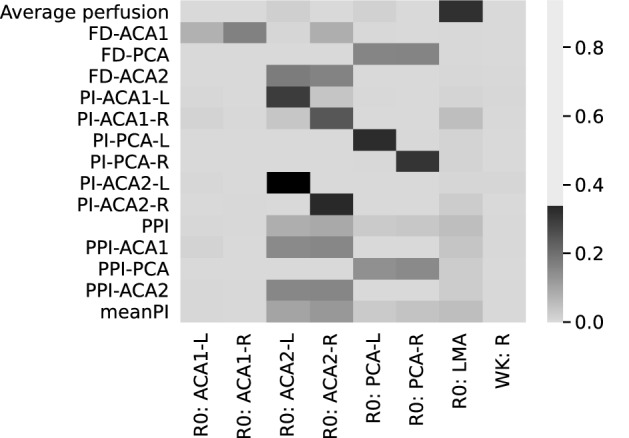
Sobol indices in case of 40% uncertainty on input parameters (on the x axis). “R0: Vx” indicates the radius of vessel Vx, “WK: R” is the resistive part of the windkessel element. Darker colours signify higher influence of the inputs on the outputs (on the y axis). The average perfusion is significantly influenced by the radius of LMAs. With high level of uncertainty on the inputs the biomarkers do not depend on R0: LMA and thus cannot be used as proxy measurements.

**Figure 6:**
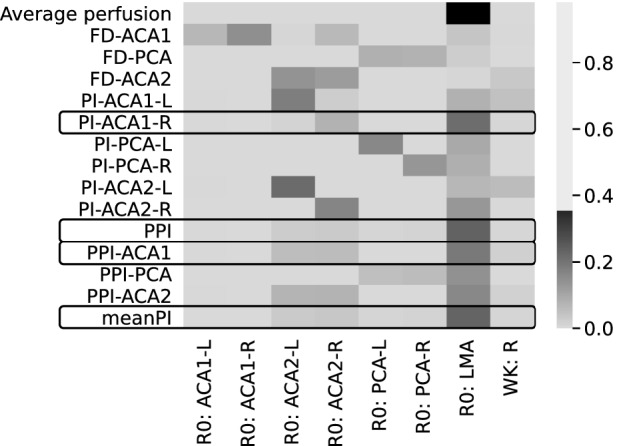
Sobol indices in case of 10% uncertainty on left and right R0: ACA2 and R0: PCA. Uncertainty on remaining parameters is 40%. Increased knowledge of ACA2 and PCA radii causes an increase in the dependency of the biomarkers on the LMA radii. The four biomarkers whose Sobol index with R0: LMA is above 0.5 are highlighted.

The Sobol indices obtained for the reduced model in the scenarios of 40% and 10% uncertainty are presented in Figs. 5 and 6 respectively as a heatmap where darker colours signify higher influence. The average perfusion is predominantly influenced by the radius of the pial collaterals (R0: LMA), as indicated by its Sobol index of 0.88. Conversely, all the proposed biomarkers show a weak dependency on the LMA radius (Sobol indices below 0.14) and are mostly influenced by the radii of PCA and ACA2.

The situation changes when variability on the radii of PCA and ACA2 is reduced to 10%. All biomarkers show a decrease of the Sobol indices associated with ACA2 and PCA, and a simultaneous increment in the influence of LMAs and distal windkessel parameters. Four biomarkers present a Sobol index greater than 0.5 (PI-ACA1-R: 0.6; PPI: 0.65; PI-ACA1: 0.53; meanPI: 0.64).

### Correlation Study

**Figure 7:**
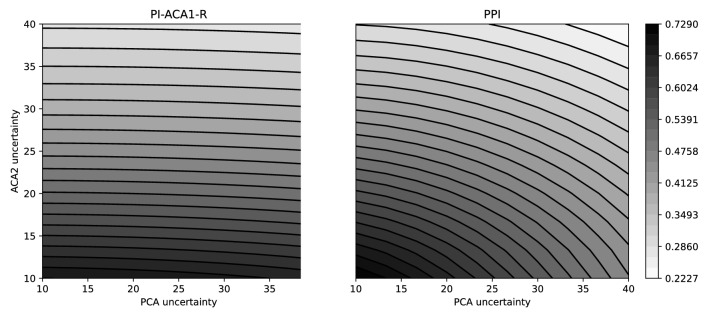
Contour plots of correlation surfaces for biomarkers PI-ACA1-R (left) and PPI (right), obtained with uncertainties on radii of PCA and ACA2 ranging from 10% to 40%.

We present here only the results for two biomarkers, pulsatility index of right ACA1 (PI-ACA1-R) and the product of all pulsatility indices (PPI). The remaining two biomarkers have similar qualitative behaviour and are reported in the Supplementary Material. Figure 7 shows the absolute value of the linear correlation coefficient between each biomarker and the level of distal perfusion for different levels of uncertainty on the parameters that can be clinically measured, the radii of ACA2 and PCA.

Lower values of uncertainties are associated with higher correlation, while lack of knowledge on the anatomy of the network yields lower predictive ability. The two biomarkers present comparable ranges of correlation coefficient but behave differently with respect to the uncertainty levels. PI-ACA1-R shows weak dependence on the PCA radius, with maximum values of $R = 0.69$ attained for 10% uncertainty on ACA2 radius. The correlation drops to $R = 0.27$ when the uncertainty on ACA2 grows.

For PPI the case is not as polarised. The maximum correlation is $R = 0.72$, which degrades to $R = 0.22$ when the uncertainty grows, although with a smaller gradient compared to the previous two biomarkers.

### Biomarker Threshold for Decision Making

**Figure 8:**
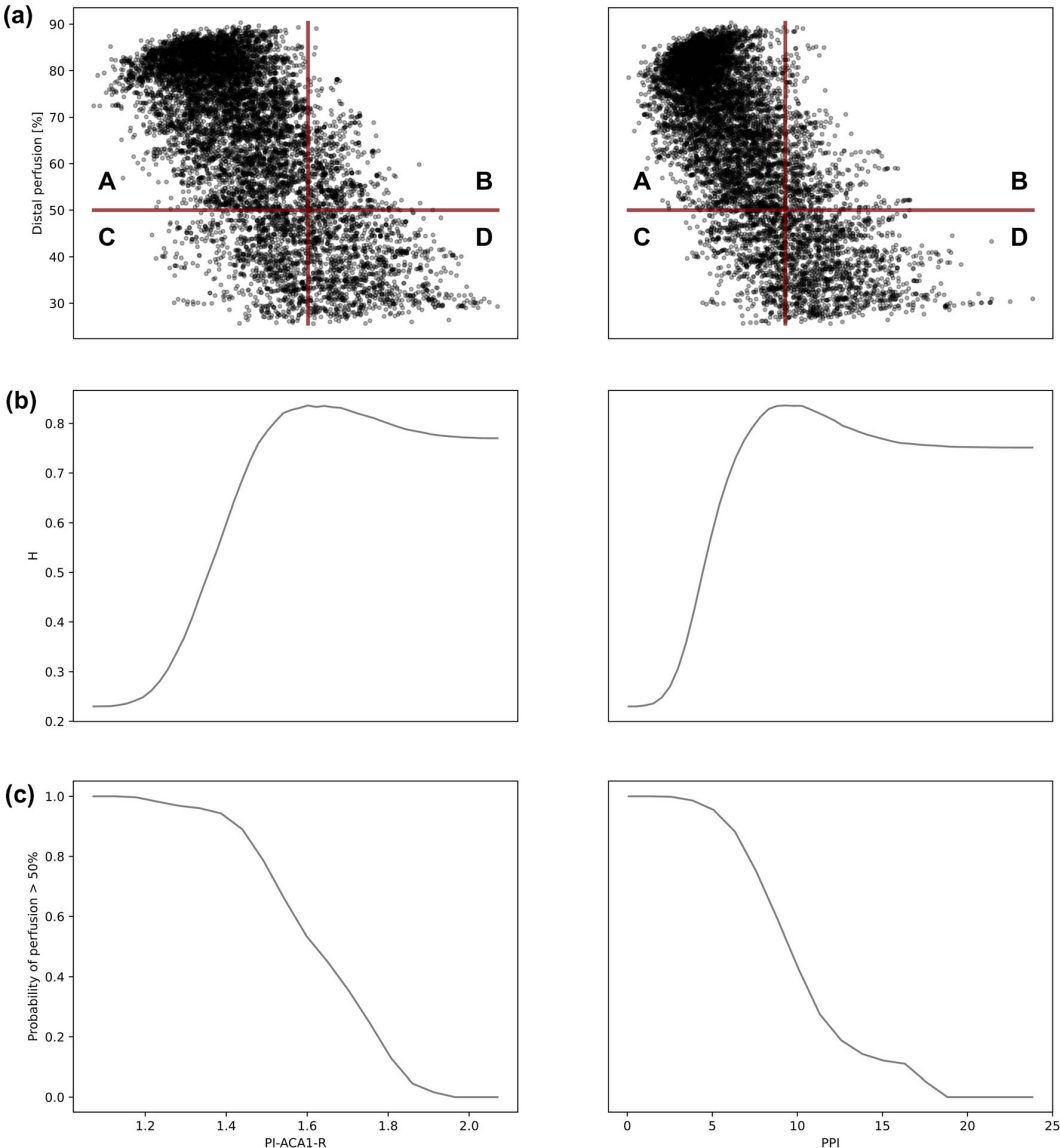
(a) Scatter plot of the distal perfusion as a function of the biomarkers. The perfusion is represented as a percentage of the healthy case. The biomarker PI-ACA1-R is on the left, while PPI on the right. The red horizontal line indicates a perfusion level of 50%, red vertical line indicates the biomarker threshold. Patients in A do not need immediate intervention and are classified correctly. Patients in B do not need immediate intervention and are classified incorrectly. Patients in C need immediate intervention and are classified incorrectly. Patients in D need immediate intervention and are classified correctly. (b) H ratio for biomarkers PI-ACA1-R (left) and PPI (right). The value of the biomarker that maximises H is chosen as the biomarker threshold. (c) Probability of perfusion >50% as a function of the observed biomarkers. Left: PI-ACA1-R. Right: PPI.

Figure 8(a) shows the scatter plots drawn from 10000 virtual patients generated by the emulator in the case of least uncertainty, corresponding to points in the bottom left part of the correlation surfaces in Fig. 7. For both biomarkers, higher perfusion is associated with lower values of the biomarkers.

The behaviour of the ratio $H$, whose maximum identifies the optimal biomarker threshold, is plotted in Fig. 8(b). Values of the threshold are PI-ACA1-R=1.6 and PPI=9.3. Perfusion probabilities are plotted in Fig. 8(c). For example, with measurement PPI=6 the patient is classified as not in need of priority treatment, with a 90% likelihood of perfusion>50%. A value PPI=15 is associated to immediate care, and a 12% probability of good perfusion.

## Discussion

We have presented a mechanistic 1D model of the leptomeningeal collaterals and developed a methodology for identifying biomarkers for distal perfusion that could aid clinical decision making.

The mechanistic model of LMAs shows features of the brain circulation that have been observed in clinical studies on stroke patients. In healthy conditions the circulation between right and left hemispheres is balanced, with nearly identical values of pressure, blood velocity and flowrate on homologous vessels on both sides. In this situation the LMAs are subject to small pressure gradients and perfused with little amount of blood. Following MCA occlusion, changes in blood pressure on the occluded side force the blood to fill the LMAs and reach distal MCA regions. This is consistent with theories on the physiology of LMAs^9,20,36^ and findings from computational studies.^25,27,28^ Our baseline model shows values of FD and PI well aligned with the literature.^17,33^ Flow velocity increases in ipsilateral ACA and PCA because of the negligible variations in their radius, combined with the reduction of distal resistance caused by the recruitment of LMAs. The drop in resistance also influences a decrease in pulsatility index,^20^ although it’s been suggested that other factors contribute to the determination of the pulsatility index.^6^ The contralateral side is only marginally affected by the occlusion, coherently with predictions from the study of Jozsa and colleagues.^16^

SSA determined that the radius of LMAs is responsible for approximately 90% of distal perfusion’s variability. According to Poiseuille’s law, the resistance to flow offered by a tube is inversely proportional to the fourth power of its radius.^9^ Consequently, variations of LMA radius between ±40% yield variations in vascular resistance between 25% and 800% of the baseline value, which is reflected on the distal perfusion because the LMAs connect the ACA2 and PCA regions with the distal branches of MCA and perfuse them when the MCA is occluded. Furthermore, the percentage variation of vascular resistance induced by variation of peripheral windkessel resistance is one order of magnitude smaller than that induced by variation of LMA radius, which explains why their Sobol indices are small. The important role of LMA radius finds indirect confirmation in clinical and computational studies. Using CT angiography, Yeo and colleagues showed that good collaterals are associated with significant recanalisation and positive outcome post pharmacological treatment.^43^ Other researchers confirmed these findings.^34,37^ In their computational. study, Padmos and colleagues showed that increasing the number of LMAs strengthens the connection between different brain regions and has the overall effect of reducing the resistance between the regions downstream the occlusion, improving exponentially the transport properties of a contrast medium across an occlusion.^25^ This is equivalent to the reduction in resistance observed in our model when the LMAs are widened.

We identified four biomarkers, all potentially measurable through TCD measurements, that can inform on the perfusion state. Flow diversion of ACA and PCA is routinely used for assessing the presence of a stroke and the degree of collateralisation^17,33,45^ but its Sobol indices show that it can’t be used as a proxy for distal MCA perfusion. On the other hand, combinations of pulsatility indices of ipsilateral and contralateral ACA1, ACA2 and PCA showed a significant dependency on the radius of LMAs. Wide or narrow collaterals induce low or high distal resistance, which in turn translates to high or low pulsatility respectively. At the same time, the quality of the collaterals affects the level of perfusion, which essentially depends on the radius of LMAs. This explains the dependency of PI-based biomarkers on the LMA radius.

Despite being counterintuitive that PI-ACA1-R, measured on the right side of the brain, is a biomarker for the perfusion in the left side during a stroke while PI-ACA1-L is not, this is explained by the fact that an ischaemic event introduces imbalance in the network and triggers phenomena of flow redistribution that breaks the symmetric behaviour characteristic of the healthy circulation. This results in PI-ACA1-L having lower predictive power than its counterpart, as also shown by its relatively low Sobol index with the perfusion (0.23).

The biomarkers showed negative correlation with the distal perfusion, with values of correlation ranging from 0.22 to 0.73 depending on the level of uncertainty on the radii of ACA2 and PCA. Existing literature confirms this weak relationship: Uzuner and colleagues^39^ identified positive correlation between the pulsatility indices on the lesion side and the National Institute of Health Stroke Scale (NIHSS). Low values of NIHSS, associated with better patient’s conditions, were attained at low values of pulsatility, which qualitatively validates our findings. The low correlation coefficient predicted by our model prevents the use of the biomarkers to univocally estimate the perfusion, but we could identify thresholds for separating the patients into binary categories. Such categories indicate whether a patient is most likely to require immediate intervention or not and are designed to optimize the management of a large patient population. Their use combined with the perfusion probabilities can help in estimating the perfusion state and guiding clinical management. Clinical studies have not focussed on the association between TCD based metrics and perfusion but have used metrics such the intracranial pressure (ICP) or the cerebral perfusion pressure (CPP) as endpoints. Bellner and colleagues^3^ identified a strong association between pulsatility index and ICP in patient with head injuries. Zweifel and coworkers^46^ confirmed these results and noted a negative correlation with CPP. However, the overall conclusion of their study was that PI should not be used as precise indicator of CPP but should rather be used as a support to other diagnostic tools. They estimated the likelihood of the patients having ICP and CPP within specific ranges, concluding that higher PI is associated to overall poor conditions. This does not directly validate our results because they studied ICP and CCP and not perfusion, but it is an indication that our model is able to realistically capture important features of the brain circulation.

Of the two biomarkers presented here, PI-ACA1-R is calculated from measurement performed on right ACA1 only, while PPI requires measurements on six different vessels. A functional use of PPI relies on a relatively precise knowledge of the radii of both ACA2 and PCA, potentially hard to acquire in a context of clinical emergency. PI-ACA1-R instead requires the determination of the radius of ipsilateral ACA2 and of the blood velocity in contralateral ACA1, which might be preferable in a clinical scenario.

This study has a number of limitations. Besides qualitative validation against literature data, we did not benchmark the biomarkers on *in vivo* data. The collection of the data necessary for *in vivo* validation of this model would need to be performed during the management of stroke patients within hospital settings. This is typically an emergency situation where disruption to standard clinical protocols has a significant impact on patient outcomes. The first step towards clinical translation of these results is their application to *in vivo* animal models. Our model did not explicitly represent brain autoregulation, which is an important feature of the cerebral physiology.^26^ Modification of distal haemodynamic conditions were implicitly represented through sampling of the windkessel parameters, but they described a population of patients rather than changes within a specific individual. Also, we did not model how tissue damage evolves over time.^31^ This is fundamental for long term evaluation of the status of brain circulation but plays a minor role in case of patient evaluation performed within few hours from the onset of the ischaemic event, which is the scenario our model is primarily concerned with. Another limitation is the extension of the network: existing studies in the literature have adopted representations of the brain circulation with thousands of vessels but limited their analysis to steady scenarios.^25^ In our case the limited extension of the network limited the computational effort and allowed a transient description of the cardiac cycle. This led to the identification of biomarkers associated to maximum, average and minimum values of the velocities, not possible with steady models.

In conclusion, our mechanistic model realistically describes the collateral circulation involved in MCA occlusion, and our hybrid mechanistic-statistical approach could identify a number of biomarkers than could potentially assist doctors in the preliminary evaluation of stroke patients.

## Supplementary Information

Below is the link to the electronic supplementary material.

Supplementary file1 (DOCX 13820 kb)
