# Supplementary material for: Determining Clinically-Viable Biomarkers for Ischaemic Stroke Through a Mechanistic and Machine Learning Approach
Source: Ann Biomed Eng. 2022 Apr 1;50(6):740–50. doi: 10.1007/s10439-022-02956-7 (PMC9079032; doi:10.1007/s10439-022-02956-7)
Supplement: Supplementary file 1 — Supplementary file1 (DOCX 13820 kb) [file 10439_2022_2956_MOESM1_ESM.docx]

**Supplementary material for:**

**Determining clinically-viable biomarkers for ischaemic stroke through a mechanistic and machine learning approach**

Ivan Benemerito^1,2^, Ana Paula Narata^3^, Andrew Narracott^1,4^, Alberto Marzo^1,2^

^1^INSIGNEO Institute for *in silico* medicine, The University of Sheffield, UK

^2^Department of Mechanical Engineering, The University of Sheffield, UK

^3^Department of Neuroradiology, University Hospital of Southampton, UK

^4^Department of Infection, Immunity and Cardiovascular Diseases, The University of Sheffield, UK

**MATERIALS AND METHODS**

*Baseline mechanistic model*

The full network for healthy patient is represented in Figure 1. Length, radii, Young’s moduli and windkessel parameters are reported in Table 1.


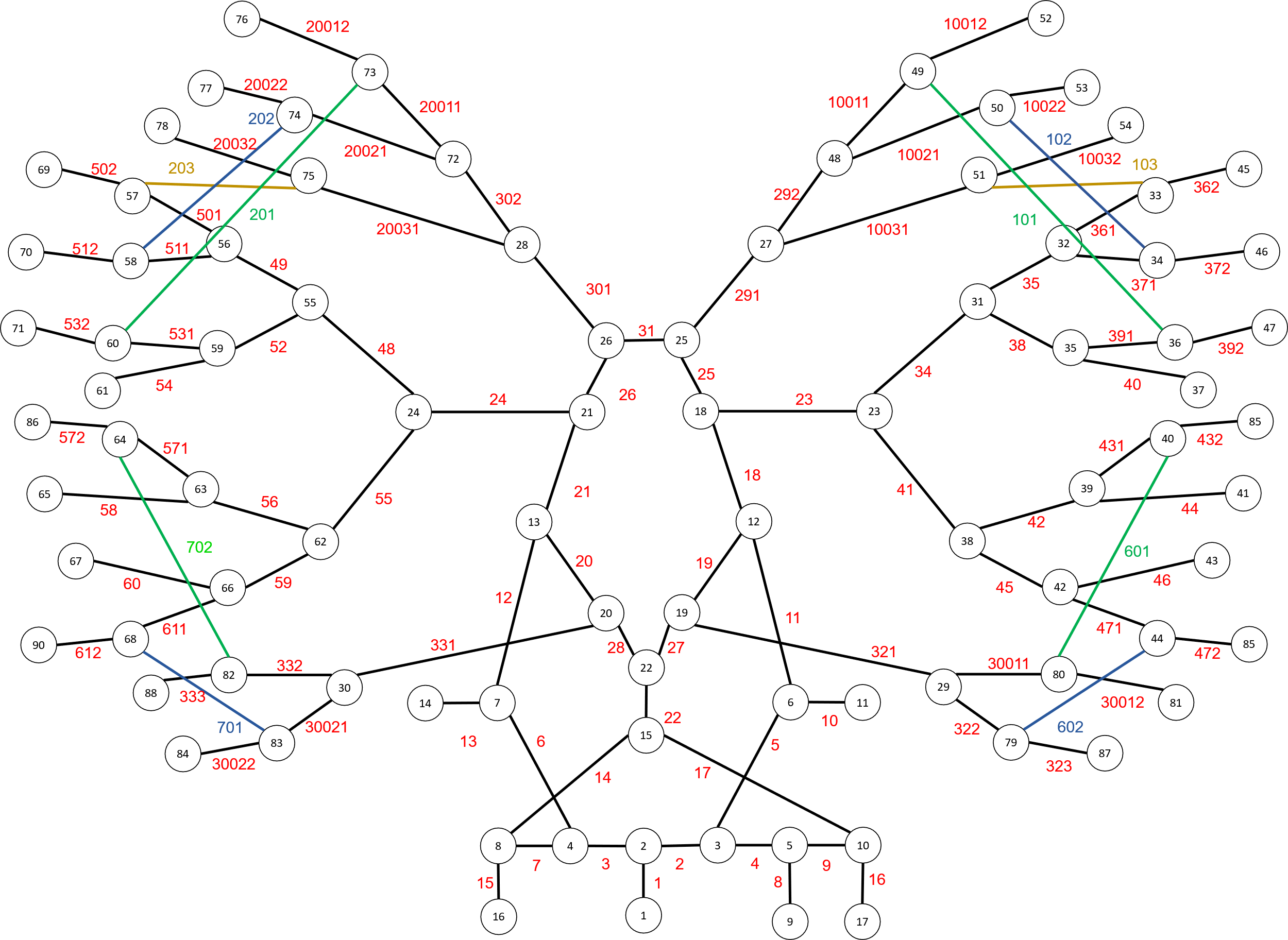


Figure 1: The network for healthy subject. Main arteries are indicated by the black lines and numbered in red. Leptomeningeal collaterals are identified by blue, yellow and green lines and numbered in the same colour.

Table 1: Structural properties of the vessels in the network.

| Name | *L* (cm) | *R_0_* (mm) | *E* (kPa) | *R_1_+R_2_ (10^9^ Pa* ·*s* ·*m^-3^)* | | Cc (10^-10^ m^3^ ·Pa^-1^) | |
| --- | --- | --- | --- | --- | --- | --- | --- |
| 1-Ascendingaorta | 4.0 | 12.0 | 400 |  | |  | |
| 2-Aorticarchi | 2.0 | 11.2 | 400 |  |  | |  |
| 3-Brachiocephalic | 3.4 | 6.2 | 400 |  | |  | |
| 4-Aorticarchii | 3.9 | 10.7 | 400 |  | |  | |
| 5-L-Commoncarotid | 20.8 | 2.5 | 400 |  | |  | |
| 6-R-Commoncarotid | 17.7 | 2.5 | 400 |  | |  | |
| 7-R-Subclavian | 3.4 | 4.23 | 400 |  | |  | |
| 8-Thoracicaorta | 15.6 | 9.8 | 400 | 0.18 | | 3.87 | |
| 9-L-Subclavian | 3.4 | 4.23 | 400 |  | |  | |
| 10-L-Ext-Carotid | 17.7 | 1.5 | 800 | 5.43 | | 1.27 | |
| 11-L-Int-Carotidi | 17.7 | 2.0 | 800 |  | |  | |
| 12-R-Int-Carotidi | 17.7 | 2.0 | 800 |  | |  | |
| 13-R-Ext-Carotid | 17.7 | 1.5 | 800 | 5.43 | | 1.27 | |
| 14-R-Vertebral | 14.8 | 1.36 | 800 |  | |  | |
| 15-R-Brachial | 42.2 | 4.03 | 400 | 2.68 | | 2.58 | |
| 16-L-Brachial | 42.2 | 4.03 | 400 | 2.68 | | 2.58 | |
| 17-L-Vertebral | 14.8 | 1.36 | 800 |  | |  | |
| 18-L-Int-Carotidii | 0.5 | 2.0 | 1600 |  | |  | |
| 19-L-Pcoa | 1.5 | 0.73 | 1600 |  | |  | |
| 20-R-Pcoa | 1.5 | 0.73 | 1600 |  | |  | |
| 21-R-Int-Carotidii | 0.5 | 2.0 | 1600 |  | |  | |
| 22-Basilar | 2.9 | 1.7 | 1600 |  | |  | |
| 23-L-Mca | 11.9 | 1.43 | 1600 |  | |  | |
| 24-R-Mca | 11.9 | 1.43 | 1600 |  | |  | |
| 25-L-Aca-A1 | 1.2 | 1.17 | 1600 |  | |  | |
| 26-R-Aca-A1 | 1.2 | 1.17 | 1600 |  | |  | |
| 27-L-Pca-P1 | 0.5 | 1.07 | 1600 |  | |  | |
| 28-R-Pca-P1 | 0.5 | 1.07 | 1600 |  | |  | |
| 291-L-Aca-A2i | 5.15 | 1.2 | 1600 |  | |  | |
| 301-R-Aca-A2 | 5.15 | 1.2 | 1600 |  | |  | |
| 31-Acoa | 0.3 | 0.54 | 1600 |  | |  | |
| 321-L-Pca-P2 | 4.3 | 1.05 | 1600 |  | |  | |
| 331-R-Pca-P2 | 4.3 | 1.05 | 1600 |  | |  | |
| 34-Sup-Trunk-Mca-I-L | 6.81 | 1.13 | 2400 |  | |  | |
| 35-Sup-Trunk-Mca-Iii-L | 5.5 | 1.0 | 2400 |  | |  | |
| 361-Orb-Frontal-L | 2.55 | 0.72 | 2400 |  | |  | |
| 371-Prefrontal-L | 1.82 | 0.71 | 2400 |  | |  | |
| 38-Sup-Trunk-Mca-Iiii-L | 3.4 | 1.01 | 2400 |  | |  | |
| 391-Central-L | 1.855 | 0.7 | 2400 |  | |  | |
| 40-Angular-L | 21.0 | 0.8 | 2400 | 28.2 | | 0.17 | |
| 41-Inf-Trunk-Mca-I-L | 4.0 | 1.13 | 2400 |  | |  | |
| 42-Inf-Trunk-Mca-Iii-L | 3.56 | 1.1 | 2400 |  | |  | |
| 431-Temp-Occipital-L | 4.6 | 0.77 | 2400 |  | |  | |
| 44-Mid-Temp-L | 8.91 | 0.77 | 2400 | 30.6 | | 0.158 | |
| 45-Inf-Trunk-Mca-Iiii-L | 3.76 | 1.0 | 2400 |  | |  | |
| 46-Ant-Temp-L | 9.5 | 0.71 | 2400 | 30.6 | | 0.134 | |
| 471-Temp-Pol-L | 3.9 | 0.71 | 2400 |  | |  | |
| 362-Orb-Frontal-L | 2.55 | 0.72 | 2400 | 35 | | 0.138 | |
| 372-Prefrontal-L | 1.82 | 0.71 | 2400 | 36 | | 0.134 | |
| 392-Central-L | 1.855 | 0.7 | 2400 | 37 | | 0.13 | |
| 432-Temp-Occipital-L | 4.6 | 0.77 | 2400 | 30.6 | | 0.158 | |
| 472-Temp-Pol-L | 3.9 | 0.71 | 2400 | 30.6 | | 0.134 | |
| 292-L-Aca-A2ii | 5.15 | 1.2 | 1600 |  | |  | |
| 10011-Paracentral-L-Aca-A2 | 2.12 | 0.9 | 1600 |  | |  | |
| 10021-Polarfrontal-L-Aca-A2 | 2.45 | 1.0 | 1600 |  | |  | |
| 10031-Orbfrontal-L-Aca-A2 | 2.265 | 1.0 | 1600 |  | |  | |
| 10012-Paracentral-L-Aca-A2 | 2.12 | 0.9 | 1600 | 31.6 | | 0.198 | |
| 10022-Polarfrontal-L-Aca-A2 | 2.45 | 1.0 | 1600 | 25.6 | | 0.31 | |
| 10032-Orbfrontal-L-Aca-A2 | 2.265 | 1.0 | 1600 | 25.6 | | 0.31 | |
| 101-Lma-Aca-Mca-I-L | 0.52 | 0.34 | 2400 |  | |  | |
| 102-Lma-Aca-Mca-Ii-L | 0.52 | 0.34 | 2400 |  | |  | |
| 103-Lma-Aca-Mca-Iii-L | 0.52 | 0.34 | 2400 |  | |  | |
| 48-Sup-Trunk-Mca-I-R | 6.81 | 1.13 | 2400 |  | |  | |
| 49-Sup-Trunk-Mca-Iii-R | 5.5 | 1.0 | 2400 |  | |  | |
| 501-Orb-Frontal-R | 2.55 | 0.72 | 2400 |  | |  | |
| 511-Prefrontal-R | 1.82 | 0.71 | 2400 |  | |  | |
| 52-Sup-Trunk-Mca-Iiii-R | 3.4 | 1.01 | 2400 |  | |  | |
| 531-Central-R | 1.855 | 0.7 | 2400 |  | |  | |
| 54-Angular-R | 21.0 | 0.8 | 2400 | 28.2 | | 0.17 | |
| 55-Inf-Trunk-Mca-I-R | 4.0 | 1.13 | 2400 |  | |  | |
| 56-Inf-Trunk-Mca-Iii-R | 3.56 | 1.1 | 2400 |  | |  | |
| 571-Temp-Occipital-R | 4.6 | 0.77 | 2400 |  | |  | |
| 58-Mid-Temp-R | 8.91 | 0.77 | 2400 | 30.6 | | 0.158 | |
| 59-Inf-Trunk-Mca-Iiii-R | 3.76 | 1.0 | 2400 |  | |  | |
| 60-Ant-Temp-R | 9.5 | 0.71 | 2400 | 30.6 | | 0.134 | |
| 611-Temp-Pol-R | 3.9 | 0.71 | 2400 |  | |  | |
| 502-Orb-Frontal-R | 2.55 | 0.72 | 2400 | 35 | | 0.138 | |
| 512-Prefrontal-R | 1.82 | 0.71 | 2400 | 36 | | 0.134 | |
| 532-Central-R | 1.855 | 0.7 | 2400 | 37 | | 0.13 | |
| 572-Temp-Occipital-R | 4.6 | 0.77 | 2400 | 30.6 | | 0.158 | |
| 612-Temp-Pol-R | 3.9 | 0.71 | 2400 | 30.6 | | 0.134 | |
| 302-R-Aca-A2ii | 5.15 | 1.2 | 1600 |  | |  | |
| 20011-Paracentral-R-Aca-A2 | 2.12 | 0.9 | 1600 |  | |  | |
| 20021-Polarfrontal-R-Aca-A2 | 2.45 | 1.0 | 1600 |  | |  | |
| 20031-Orbfrontal-R-Aca-A2 | 2.265 | 1.0 | 1600 |  | |  | |
| 20012-Paracentral-R-Aca-A2 | 2.12 | 0.9 | 1600 | 31.6 | | 0.198 | |
| 20022-Polarfrontal-R-Aca-A2 | 2.45 | 1.0 | 1600 | 25.6 | | 0.31 | |
| 20032-Orbfrontal-R-Aca-A2 | 2.265 | 1.0 | 1600 | 25.6 | | 0.31 | |
| 201-Lma-Aca-Mca-I-R | 0.52 | 0.34 | 2400 |  | |  | |
| 202-Lma-Aca-Mca-Ii-R | 0.52 | 0.34 | 2400 |  | |  | |
| 203-Lma-Aca-Mca-Iii-R | 0.52 | 0.34 | 2400 |  | |  | |
| 322-L-Pca-P2 | 2.15 | 1.05 | 1600 |  | |  | |
| 30011-L-Parietooccipital-I | 1.5 | 1.05 | 1600 |  | |  | |
| 30012-L-Parietooccipital-Ii | 1.5 | 1.05 | 1600 | 24 | | 0.62 | |
| 323-L-Pca-P2 | 2.15 | 1.05 | 1600 | 24 | | 0.62 | |
| 332-R-Pca-P2 | 2.15 | 1.05 | 1600 |  | |  | |
| 30021-R-Parietooccipital-I | 1.5 | 1.05 | 1600 |  | |  | |
| 30022-R-Parietooccipital-Ii | 1.5 | 1.05 | 1600 | 24 | | 0.62 | |
| 333-R-Pca-P2 | 2.15 | 1.05 | 1600 | 24 | | 0.62 | |
| 601-Lma-Pca-Mca-I-L | 0.52 | 0.34 | 2400 |  | |  | |
| 701-Lma-Pca-Mca-I-R | 0.52 | 0.34 | 2400 |  | |  | |
| 602-Lma-Pca-Mca-Ii-L | 0.52 | 0.34 | 2400 |  | |  | |
| 702-Lma-Pca-Mca-Ii-R | 0.52 | 0.34 | 2400 |  | |  | |

*Emulator validation*

Emulator trained on 900 points and validated on 200. Figure 2 shows the scatter plot of simulated against emulated outputs after the validation of the emulator.


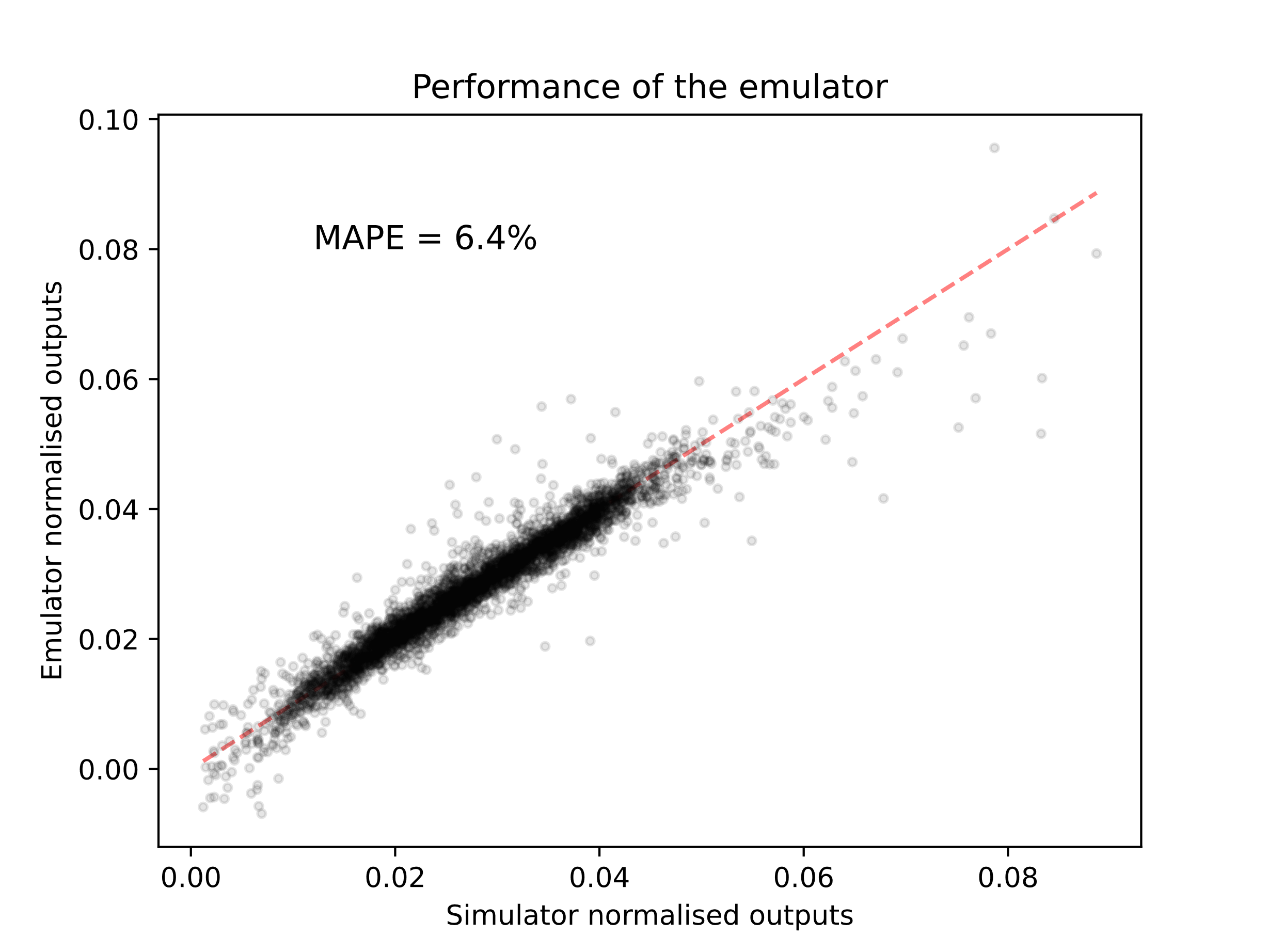


Figure 2: Validation of the emulator. On the x axis are reported the normalised values of the simulated outputs, on the y axis the normalised value of the simulator. Perfect emulator fit is represented by the red dashed line.

*Sobol’s Sensitivity Analysis on full parameter space*

Heatmaps for Sobol’s sensitivity analysis on full parameter space are shown in Figure 3.
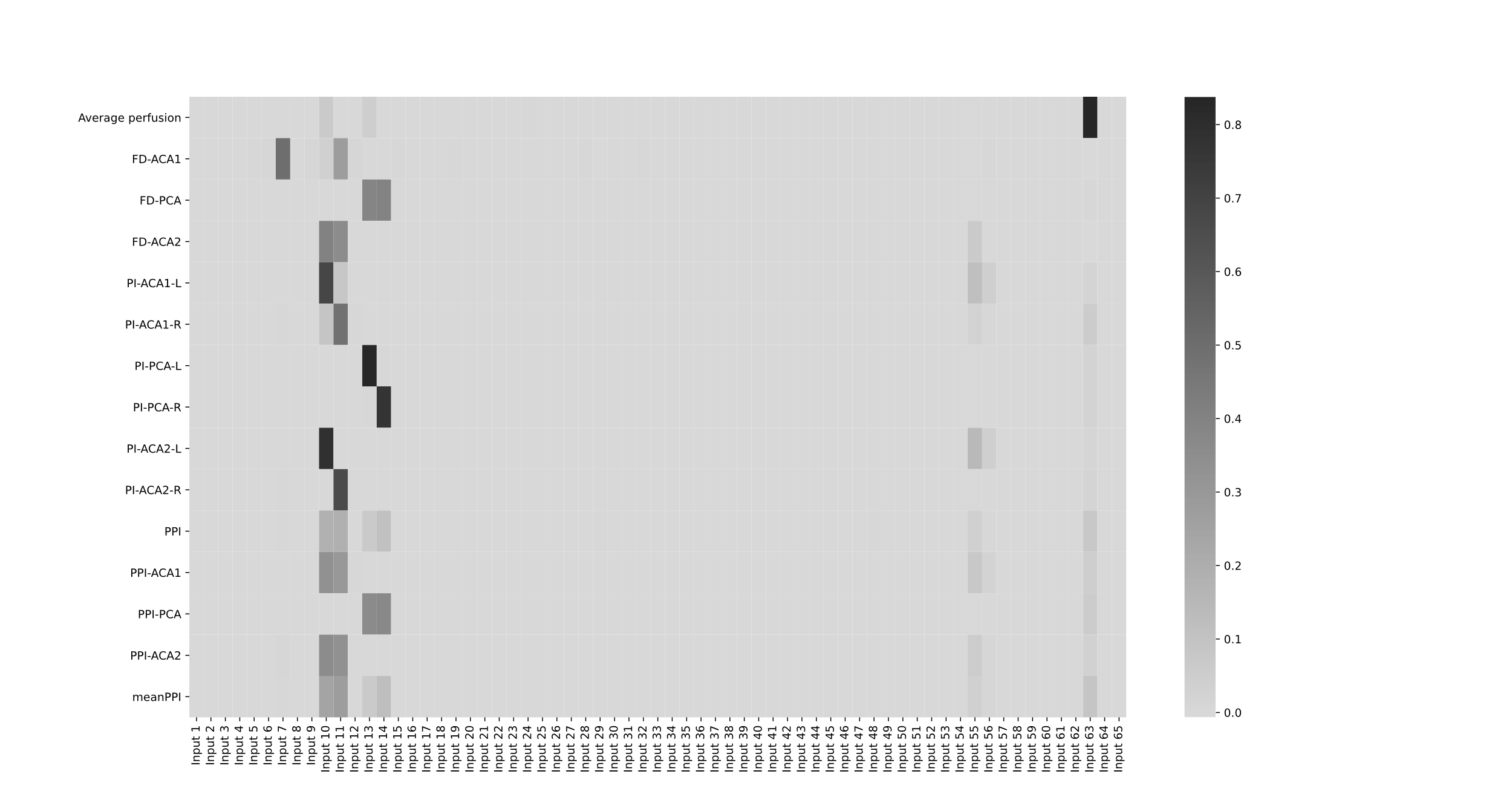

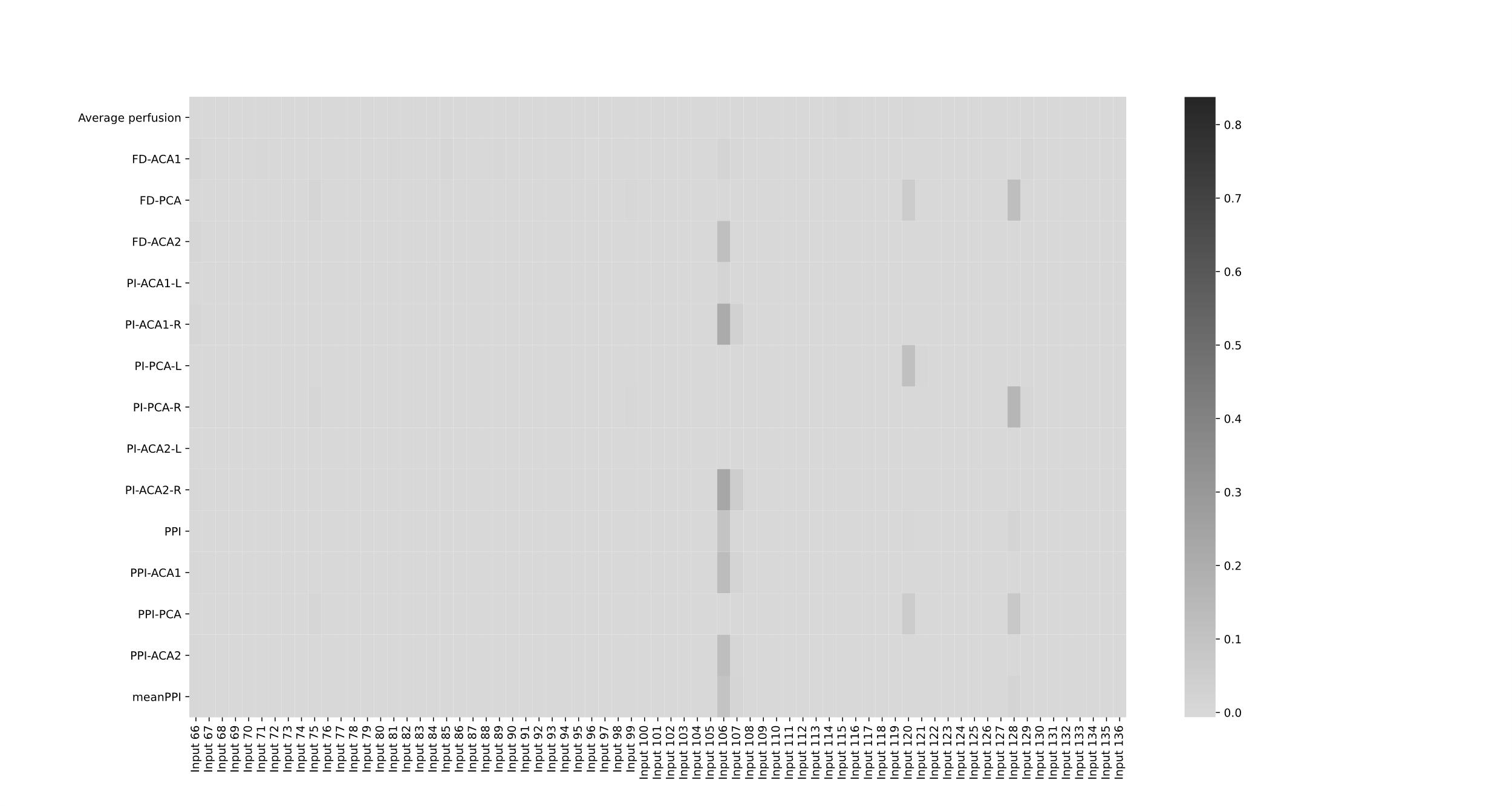


Figure 3: Heatmap of Sobol indices for sensitivity analysis on the full parameter space. Input names are reported in Table 2.

Table 2: Input names for 1 sensitivity analysis.

| Inputs 1 to 34 | Inputs 35 to 68 | Inputs 69 to 102 | Inputs 103 to 136 |
| --- | --- | --- | --- |
| Input 1: 18_R0 | Input 35: 362_R0 | Input 69: 511_R0 | Input 103: 20021_R0 |
| Input 2: 19_R0 | Input 36: 362_R1 | Input 70: 52_R0 | Input 104: 20031_R0 |
| Input 3: 20_R0 | Input 37: 362_Cc | Input 71: 531_R0 | Input 105: 20012_R0 |
| Input 4: 21_R0 | Input 38: 372_R0 | Input 72: 54_R0 | Input 106: 20012_R1 |
| Input 5: 24_R0 | Input 39: 372_R1 | Input 73: 54_R1 | Input 107: 20012_Cc |
| Input 6: 25_R0 | Input 40: 372_Cc | Input 74: 54_Cc | Input 108: 20022_R0 |
| Input 7: 26_R0 | Input 41: 392_R0 | Input 75: 55_R0 | Input 109: 20022_R1 |
| Input 8: 27_R0 | Input 42: 392_R1 | Input 76: 56_R0 | Input 110: 20022_Cc |
| Input 9: 28_R0 | Input 43: 392_Cc | Input 77: 571_R0 | Input 111: 20032_R0 |
| Input 10: 291_R0 | Input 44: 432_R0 | Input 78: 58_R0 | Input 112: 20032_R1 |
| Input 11: 301_R0 | Input 45: 432_R1 | Input 79: 58_R1 | Input 113: 20032_Cc |
| Input 12: 31_R0 | Input 46: 432_Cc | Input 80: 58_Cc | Input 114: 201_R0 |
| Input 13: 321_R0 | Input 47: 472_R0 | Input 81: 59_R0 | Input 115: 202_R0 |
| Input 14: 331_R0 | Input 48: 472_R1 | Input 82: 60_R0 | Input 116: 203_R0 |
| Input 15: 34_R0 | Input 49: 472_Cc | Input 83: 60_R1 | Input 117: 322_R0 |
| Input 16: 35_R0 | Input 50: 292_R0 | Input 84: 60_Cc | Input 118: 30011_R0 |
| Input 17: 361_R0 | Input 51: 10011_R0 | Input 85: 611_R0 | Input 119: 30012_R0 |
| Input 18: 371_R0 | Input 52: 10021_R0 | Input 86: 502_R0 | Input 120: 30012_R1 |
| Input 19: 38_R0 | Input 53: 10031_R0 | Input 87: 502_R1 | Input 121: 30012_Cc |
| Input 20: 391_R0 | Input 54: 10012_R0 | Input 88: 502_Cc | Input 122: 323_R0 |
| Input 21: 40_R0 | Input 55: 10012_R1 | Input 89: 512_R0 | Input 123: 323_R1 |
| Input 22: 40_R1 | Input 56: 10012_Cc | Input 90: 512_R1 | Input 124: 323_Cc |
| Input 23: 40_Cc | Input 57: 10022_R0 | Input 91: 512_Cc | Input 125: 332_R0 |
| Input 24: 41_R0 | Input 58: 10022_R1 | Input 92: 532_R0 | Input 126: 30021_R0 |
| Input 25: 42_R0 | Input 59: 10022_Cc | Input 93: 532_R1 | Input 127: 30022_R0 |
| Input 26: 431_R0 | Input 60: 10032_R0 | Input 94: 532_Cc | Input 128: 30022_R1 |
| Input 27: 44_R0 | Input 61: 10032_R1 | Input 95: 572_R0 | Input 129: 30022_Cc |
| Input 28: 44_R1 | Input 62: 10032_Cc | Input 96: 572_R1 | Input 130: 333_R0 |
| Input 29: 44_Cc | Input 63: 101_R0 | Input 97: 572_Cc | Input 131: 333_R1 |
| Input 30: 45_R0 | Input 64: 102_R0 | Input 98: 612_R0 | Input 132: 333_Cc |
| Input 31: 46_R0 | Input 65: 103_R0 | Input 99: 612_R1 | Input 133: 601_R0 |
| Input 32: 46_R1 | Input 66: 48_R0 | Input 100: 612_Cc | Input 134: 701_R0 |
| Input 33: 46_Cc | Input 67: 49_R0 | Input 101: 302_R0 | Input 135: 602_R0 |
| Input 34: 471_R0 | Input 68: 501_R0 | Input 102: 20011_R0 | Input 136: 702_R0 |

**RESULTS**

*Contour plots of correlation surface for PPI-ACA1 and meanPI*

Correlation surfaces for PPI-ACA1 and meanPI are shown in Figure 4.


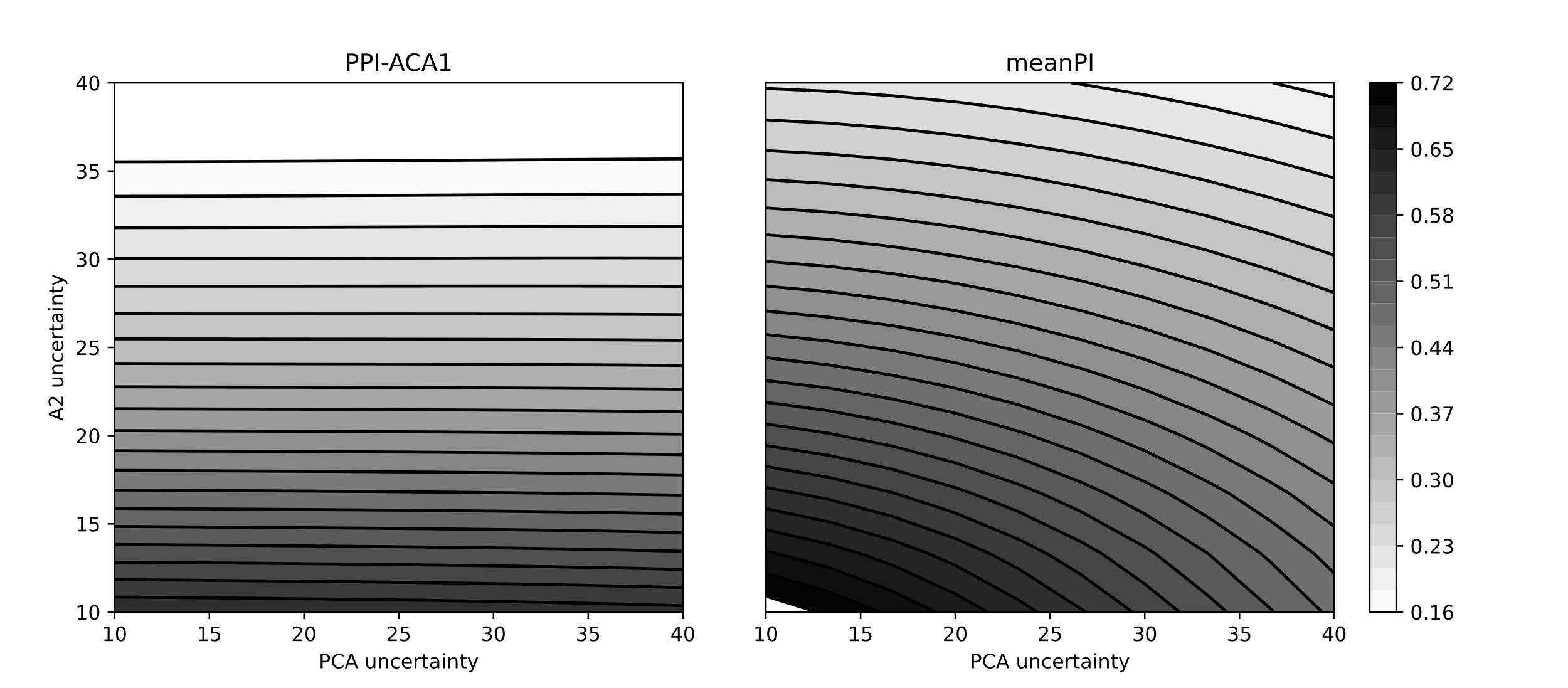


Figure 4: Contour plots of correlation surfaces for biomarkers PPI-ACA1 (left) and meanPI (right), obtained with uncertainties on radii of PCA and ACA2 ranging from 10% to 40%.

*Scatter plots, thresholds and probabilities for PPI-ACA1 and meanPI*

Scatter plot, $H$ function for threshold and perfusion probabilities for PPI-ACA1 and meanPI are plotted in Figure 5.

Threshold PPI-ACA1: 2.2

Threshold meanPPI: 1.44


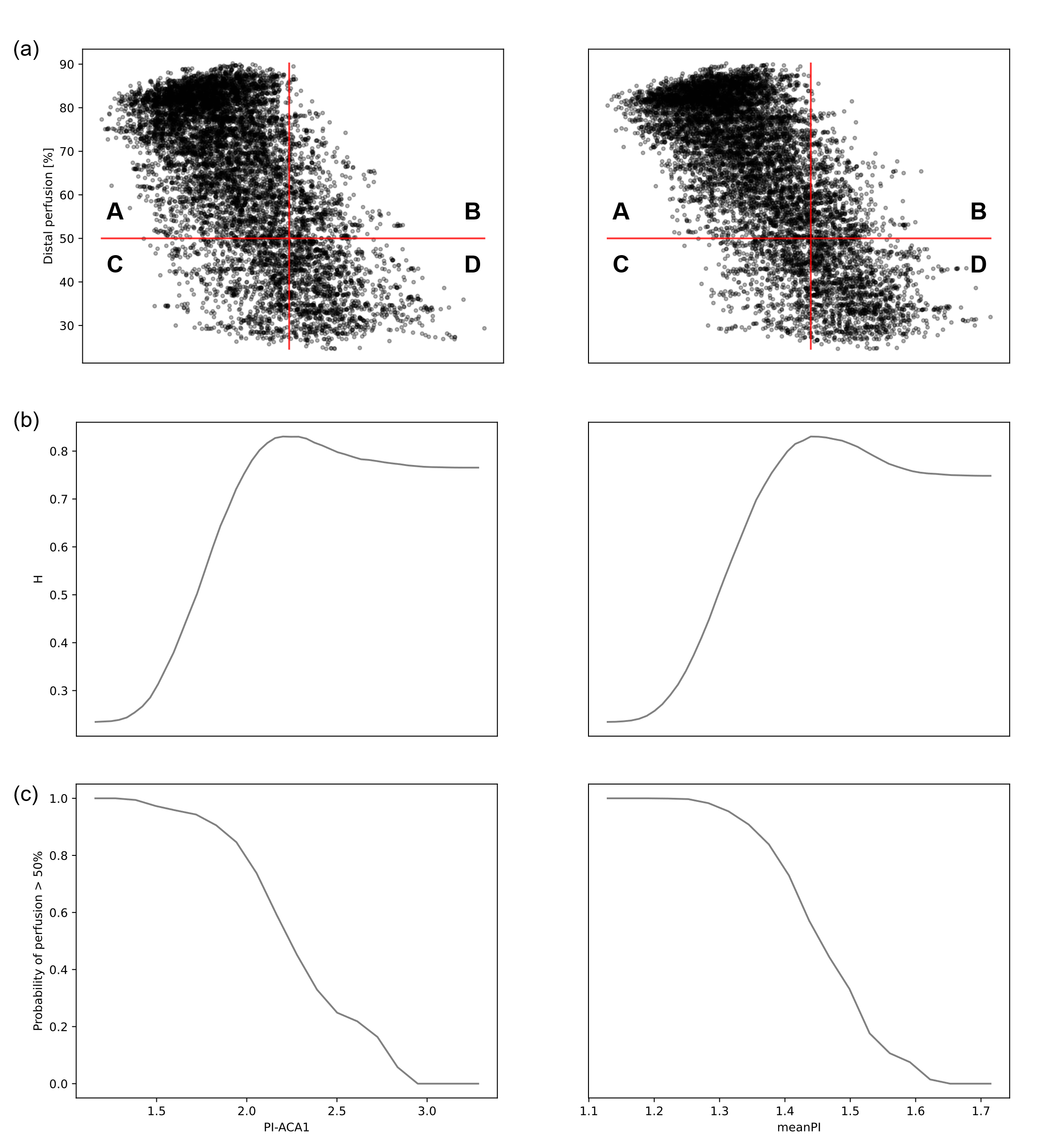


Figure 5: (a): Scatter plot of the distal perfusion as a function of the biomarkers. The perfusion is represented as a percentage of the healthy case. The biomarker PI-ACA1 is on the left, while meanPI on the right. The red horizontal line indicates a perfusion level of 50%, red vertical line indicates the biomarker threshold. Patients in A do not need immediate intervention and are classified correctly. Patients in B do not need immediate intervention and are classified incorrectly. Patients in C need immediate intervention and are classified incorrectly. Patients in D need immediate intervention and are classified correctly. (b): H ratio for biomarkers PI-ACA1 (left) and meanPI (right). The value of the biomarker that maximises H is chosen as the biomarker threshold. (c): Probability of perfusion >50% as a function of the observed biomarkers. Left: PI-ACA1. Right: meanPI.
